# Global, regional and national trends in tuberculosis incidence and main risk factors: a study using data from 2000 to 2021

**DOI:** 10.1186/s12889-023-17495-6

**Published:** 2024-01-02

**Authors:** Wentao Bai, Edward Kwabena Ameyaw

**Affiliations:** 1 School of Graduate Studies, Lingnan University, Tuen Mun, New Territories, Hong Kong; 2 L & E Research Consult Ltd, Upper West Region, Ghana

**Keywords:** Tuberculosis, Spatial autocorrelation, Risk factors, Multiple stepwise regression analysis, Autoregressive integrated moving average, Global

## Abstract

**Background:**

Despite the significant progress over the years, Tuberculosis remains a major public health concern and a danger to global health. This study aimed to analyze the spatial and temporal characteristics of the incidence of tuberculosis and its risk factors and to predict future trends in the incidence of Tuberculosis.

**Methods:**

This study used secondary data on tuberculosis incidence and tuberculosis risk factor data from 209 countries and regions worldwide between 2000 and 2021 for analysis. Specifically, this study analyses the spatial autocorrelation of Tuberculosis incidence from 2000 to 2021 by calculating Moran’s I and identified risk factors for Tuberculosis incidence by multiple stepwise linear regression analysis. We also used the Autoregressive Integrated Moving Average model to predict the trend of Tuberculosis incidence to 2030. This study used ArcGIS Pro, Geoda and R studio 4.2.2 for analysis.

**Results:**

The study found the global incidence of Tuberculosis and its spatial autocorrelation trends from 2000 to 2021 showed a general downward trend, but its spatial autocorrelation trends remained significant (Moran’s I = 0.465, P < 0.001). The risk factors for Tuberculosis incidence are also geographically specific. Low literacy rate was identified as the most pervasive and profound risk factor for Tuberculosis.

**Conclusions:**

This study shows the global spatial and temporal status of Tuberculosis incidence and risk factors. Although the incidence of Tuberculosis and Moran’s Index of Tuberculosis are both declining, there are still differences in Tuberculosis risk factors across countries and regions. Even though literacy rate is the leading risk factor affecting the largest number of countries and regions, there are still many countries and regions where gender (male) is the leading risk factor. In addition, at the current rate of decline in Tuberculosis incidence, the World Health Organization’s goal of ending the Tuberculosis pandemic by 2030 will be difficult to achieve. Targeted preventive interventions, such as health education and regular screening of Tuberculosis-prone populations are needed if we are to achieve the goal. The results of this study will help policymakers to identify high-risk groups based on differences in TB risk factors in different areas, rationalize the allocation of healthcare resources, and provide timely health education, so as to formulate more effective Tuberculosis prevention and control policies.

**Supplementary Information:**

The online version contains supplementary material available at 10.1186/s12889-023-17495-6.

## Background

Tuberculosis (TB), caused by the mycobacterium Tuberculosis complex, is a longstanding global health challenge. Its origins can be traced back 9,000 years through the detection of TB in ancient human remains [1]. TB primarily spreads through respiratory droplets released during activities such as coughing, sneezing, and talking, allowing the inhalation of Mycobacterium Tuberculosis particles by others. Additionally, the infection can occur through the mouth, intestines, and skin [2]. With approximately 25% of the global population infected with Mycobacterium Tuberculosis, new infections occur in about 1% of the population annually [3]. To combat the TB epidemic, several global strategies have been implemented. In 2018, the United Nations held a high-level meeting on TB, prioritising discussions on the pandemic and eradication strategies to the level of heads of state and government [4]. All UN member nations have pledged to strengthen efforts and eliminate TB by 2030 [5, 6]. Countries like China, India, and the United States have developed national programs and policies to prevent and control TB [4, 7, 8].

Individuals with active TB can transmit the disease to approximately 10–15 people each year through close contact [9]. Despite a net reduction of around 10% in TB incidence between 2015 and 2021, it remains a significant public health challenge globally. Worryingly, there has been a 3.9% increase in the incidence of TB between 2020 and 2021, reversing the downward trend observed for the majority of the past two decades [10]. Additionally, the emergence of drug-resistant TB, exacerbated by the misuse of antibiotics, further complicates the fight against the TB epidemic. Furthermore, the disruption of health services due to the COVID-19 pandemic has contributed to an increase in TB-related deaths worldwide between 2019 and 2021 [10, 11]. Although TB may frequently be remedied with the correct treatment plan, a significant reduction in its burden is still a distant goal for many countries [12].

The spatial distribution of TB incidence exhibits significant regional variations. Southeast Asia and Africa account for nearly 70% of all global TB cases, with the majority of high-incidence countries situated in these regions [13, 14]. Moreover, the decline in the global burden of TB has varied considerably across countries and regions. For instance, the annual percentage decline in TB incidence among HIV-negative individuals between 2006 and 2016 ranged from 6.2% in Kazakhstan to 1.2% in the Philippines [15].

The incidence of TB is influenced by various risk factors. Diabetes is a significant risk factor for active TB, with diabetic patients having a three-fold higher risk of acquiring TB compared to those without diabetes [16]. Undernourishment is also a crucial risk factor, associated with increased TB incidence, severity, poorer treatment outcomes, and higher mortality rates [17–19]. Workplace exposure to PM2.5 has been linked to smear-positive TB, as it may increase the risk of Mycobacterium TB transmission [20]. Additionally, social and economic factors, such as low socio-economic status and limited literacy, contribute to the risk of TB [21, 22]. Age is another important risk factor, with TB prevalence rates increasing significantly beyond the age of 65 [23, 24]. Besides, the incidence of TB is substantially higher in males compared to females [25–27]. Tuberculosis is also a social disease with medical aspects, it is closely related to the social factors of a country or region [28, 29].

Understanding the spatial distribution characteristics of TB incidence and the associated risk factors is essential for effective prevention and control strategies [30]. Spatial analysis can optimize resource allocation and aid in early diagnosis, transmission reduction. Consequently, this study aims to spatially investigate the global, regional and national trends in Tuberculosis incidence and the key underlying risk factors over time, thus from 2000 to 2021. This will contribute to the implementation of evidence-based and targeted tuberculosis prevention and control measures by policymakers in different countries and regions, thus assisting in achieving the global goal of ending the TB epidemic.

## Methods

### Data source

This study used secondary data for analysis. The quantitative data were obtained from World Bank Open Data. The data have been collected and compiled by the World Bank from its original sources. In order to make the study as comprehensive and complete as possible, we chose to analyze data from all years and all countries and regions included in the database, encompassing incidence of TB and TB risk factor data from 209 major countries and regions worldwide spanning the years 2000 to 2021 [10, 31–37]. The incidence of TB and different tuberculosis risk factor data have different original sources and range of data values, as indicated in Table 1. The geographical data used in this study was obtained from Natural Earth, we used geographic location information and boundary demarcation information for countries and regions from their large-scale world map data for spatial analysis.

**Table 1:** Original source and range of the data

Indicator	Original source	Range of data values
Incidence of Tuberculosis (per 100,000 people)	World Health Organization, Global Tuberculosis Report	0-1590
Literacy rate, adult total (% of people ages 15 and above)	UNESCO Institute for Statistics (UIS)	14.37604046-100
PM2.5 air pollution, mean annual exposure (micrograms per cubic meter)	Global Burden of Disease Study 2019	5.861331001–100.7844279
Population ages 65 and above (% of total population)	World Population Prospects: 2022 Revision	0.171770443–35.97012484
Population, male (% of total population)	World Population Prospects: 2022 Revision	45.02005393–76.60578262
Poverty headcount ratio at $2.15 a day (2017 PPP) (% of population)	World Bank, Poverty and Inequality Platform. Luxembourg Income Study database	0-91.5
Prevalence of undernourishment (% of population)	Food and Agriculture Organization	2.5–70.9
Diabetes prevalence (% of population ages 20 to 79)	International Diabetes Federation, Diabetes Atlas	0-30.8
GDP per capita (current US$)	World Bank national accounts data, and OECD National Accounts data files	110.4609-234315.4605

### Data standardization

In this study, we compared the magnitude of the regression coefficients to directly determine the priority of risk factors, but the range of values of each independent variable involved in this study varies widely, in order to avoid the regression coefficients will be affected by the scale of the values of each independent variable, it is necessary to do the data standardization. Since this study is based on real-world data, we wanted to preserve as much of the relationship between the original data values as possible, and in addition, since all of the data are known and no new values will be added, we chose to use the max-min normalization method for data standardization. This method facilitates the comparison of indicators of different units or magnitudes. The formula is shown below, ${x}_{caled}$ represents the normalized value, x represents the initial value.

$${x}_{caled}=\frac{x-{x}_{min}}{{x}_{max}-{x}_{min}}$$

### Missing data management

For missing data within countries and regions, we use the nearest neighbor interpolation for data that are unevenly distributed and have no obvious linear relationship, and we use linear interpolation for data that are uniformly distributed and have smoother changes. For interpolation of missing data between countries and regions, we use the inverse distance interpolation weighted (IDW) method. This method interpolates on the assumption that each location has a local impact that declines with distance. It gives more weight to the nearest point to the projected position, and the weight diminishes with distance [38]. It can model the spatial variation of variable data and this interpolation method is widely used in spatial data interpolation in fields such as public health and epidemiology.

### Data analysis

We used ArcGIS Pro and GeoDa for spatial statistical analysis and utilized RStudio 4.2.2 for regression analysis. We first cleaned and collated the data, then, spatial correlation analysis was conducted using Moran’s I index and regression analysis was used to identify the risk factors for TB by comparing regression coefficients, and finally, ARIMA models were used to predict future trends.

Moran’s I was used for the spatial analysis and it is a way of measuring spatial autocorrelation. Specifically, the global and local Moran’s I were utilized to investigate the spatial distribution characteristics of TB incidence rates. Simply said, it is a method of quantifying how tightly values are grouped together in a 2-D space [39]. Moran’s I is equivalent to the Pearson correlation coefficient in 2-D space [40]. Local Moran’s I provides for a more precise categorization of geographical clusters into four types. The value for Moran’s I can range from − 1 to 1. Positive values of Moran’s I means that neighboring areas tend to have similar values for the variable being analyzed. Negative values of Moran’s I means that surrounding areas tend to have dissimilar values for the variable being analyzed. A value of zero for Moran’s I means that the values of the variable are randomly distributed across space. The Moran’s I is often used in spatial epidemiological studies, where spatial autocorrelation between the number of diseases may reflect the true correlation between cases due to infection [41].

In this study, multiple stepwise linear regression was employed to examine the main risk factors of TB in various countries and regions. Multiple stepwise linear regression is used to minimize the AIC value as a criterion for the inclusion and exclusion of variables, avoiding over-complexity and over-fitting of the model and multicollinearity, thus improving the interpretability of the model and creating a model that simulates the real-world situation as closely as possible. We used the stepAIC function from RStudio’s MASS package for the regression analysis, and we determined the main risk factors of TB in different regions by comparing the absolute values of the regression coefficients.

ARIMA model was used to forecast future trends. ARIMA (p, d, q) is a popular time series analysis model that combines three components: autoregression (AR), differencing (I), and moving average (MA). The ‘auto.arima’ function in R is a popular tool for selecting the appropriate ARIMA model for a given time series dataset. It uses an algorithm that searches through different combinations of p, d, and q to find the best fit model based on evaluation criteria such as unit root tests, minimax AICc (Akaike Information Criterion corrected), and maximum likelihood estimation (MLE). The function can automatically determine the optimal values for p, d, and q based on these evaluation criteria [42].

## Results

### Descriptive results

**Fig. 1:**
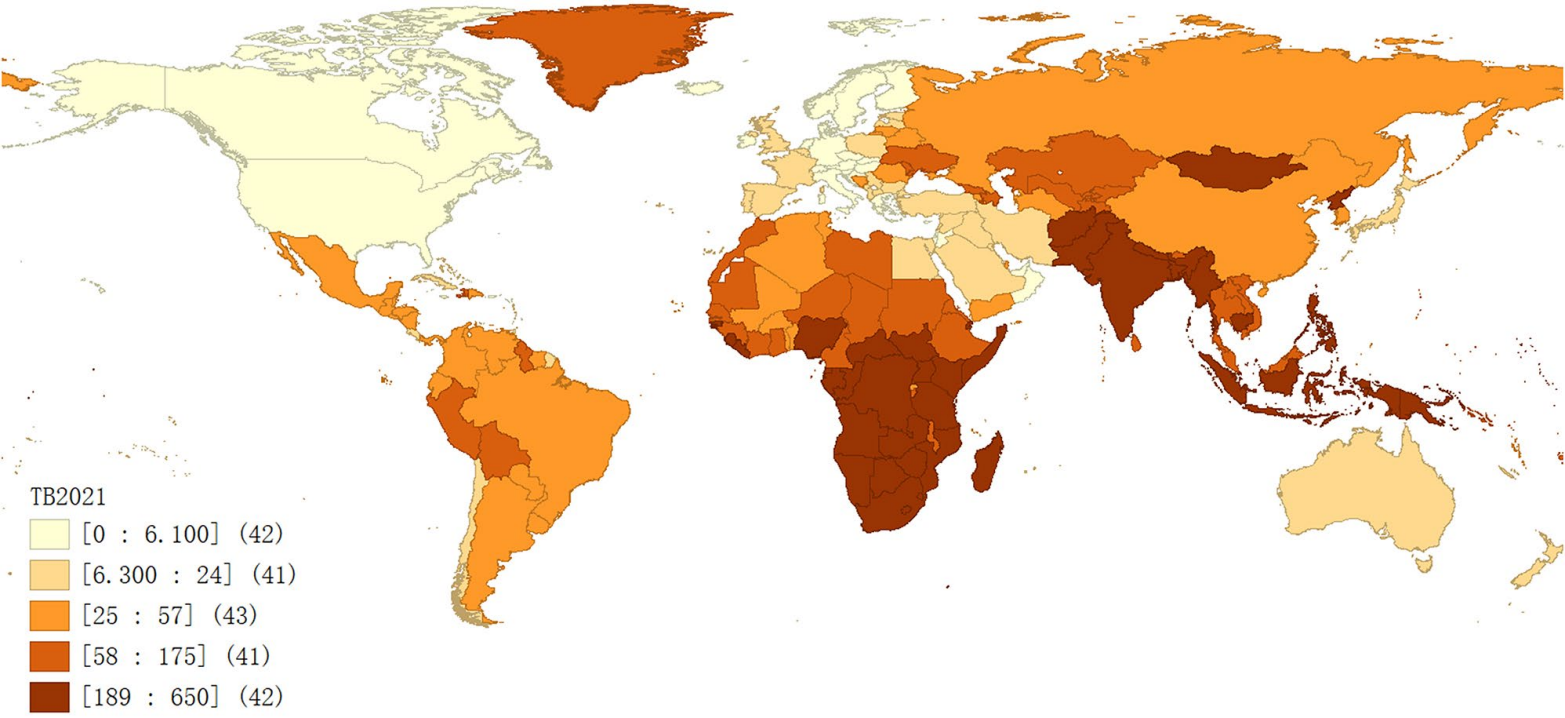
Global distribution of TB incidence in 2021

As evidenced in Fig. 1, the incidence of TB in 2021 was high in Africa, especially in Central and East Africa. In Southeast Asia and South Asia, the incidence of TB was also relatively high. The countries and regions with lower incidence of TB were mainly concentrated in North America and Europe.

**Fig. 2:**
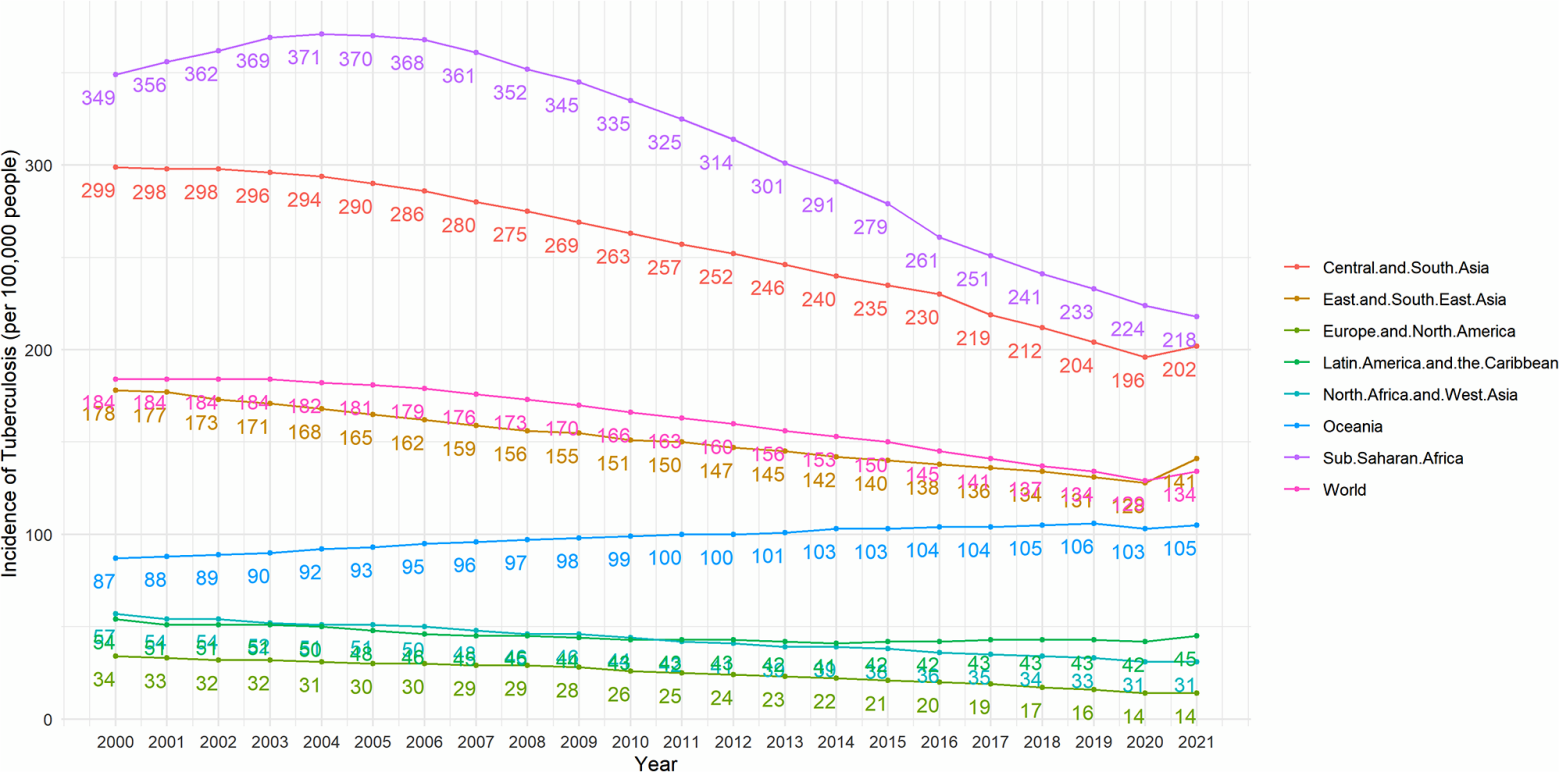
Global and region category trends in incidence of TB (2000–2021)

The regional grouping in Fig. 2 is based on the region grouping used in the United Nations 2019 Sustainable Development Goals Indicators Report [43]. From 2000 to 2021, except for Oceania, the incidence of TB exhibited a downward trend in all other areas. The greatest substantial drop was observed in sub-Saharan North Africa, while the decline in Latin America and the Caribbean was the least pronounced, and there was even a slight rebound from 2016 to 2021. TB incidence in sub-Saharan Africa and Central and South Asia has been at a high level, well above the world average, and the East and Southeast Asia TB incidence rates are closer to the world average and have similar trends (Correlation coefficient = 0.96, P < 0.05). TB incidence in Oceania, North Africa and West Asia, Europe and North America, and Latin America and the Caribbean is at a lower level compared to the world average.

**Fig. 3:**
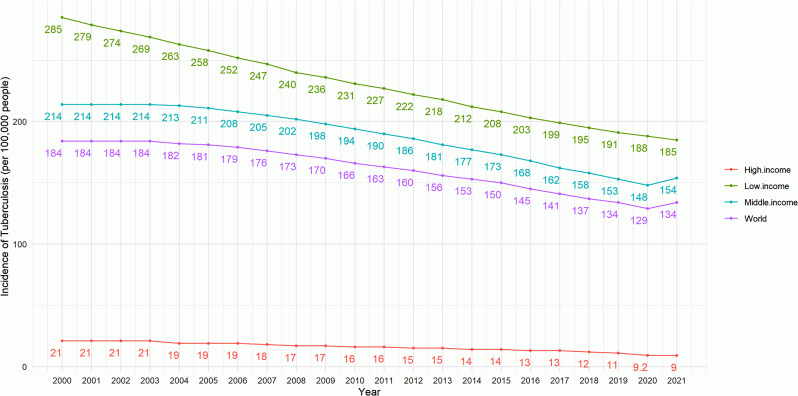
Global and income category trends in incidence of TB (2000–2021)

Income levels are categorized according to the “World Bank country classifications by income level: 2022–2023”, with Gross National Income (GNI) per capita less than 1085 USD as low income, GNI per capita greater than 1086 USD and less than 13,205 USD as middle income, and GNI per capita greater than 13,205 USD as high income. GNI per capita greater than 1086USD and less than 13205USD is classified as middle income and GNI per capita greater than 13205USD is classified as high income [44]. We can find that from 2000 to 2021, the incidence of TB in middle- and low-income countries and regions was significantly higher than that in high-income countries and regions, and also higher than the world’s average. However, the incidence of TB in middle- and low-income countries and regions showed a significant downward trend, while the incidence in high-income countries and regions did not show a significant downward trend, as shown in Fig. 3.

### Spatial analysis results

**Fig. 4:**
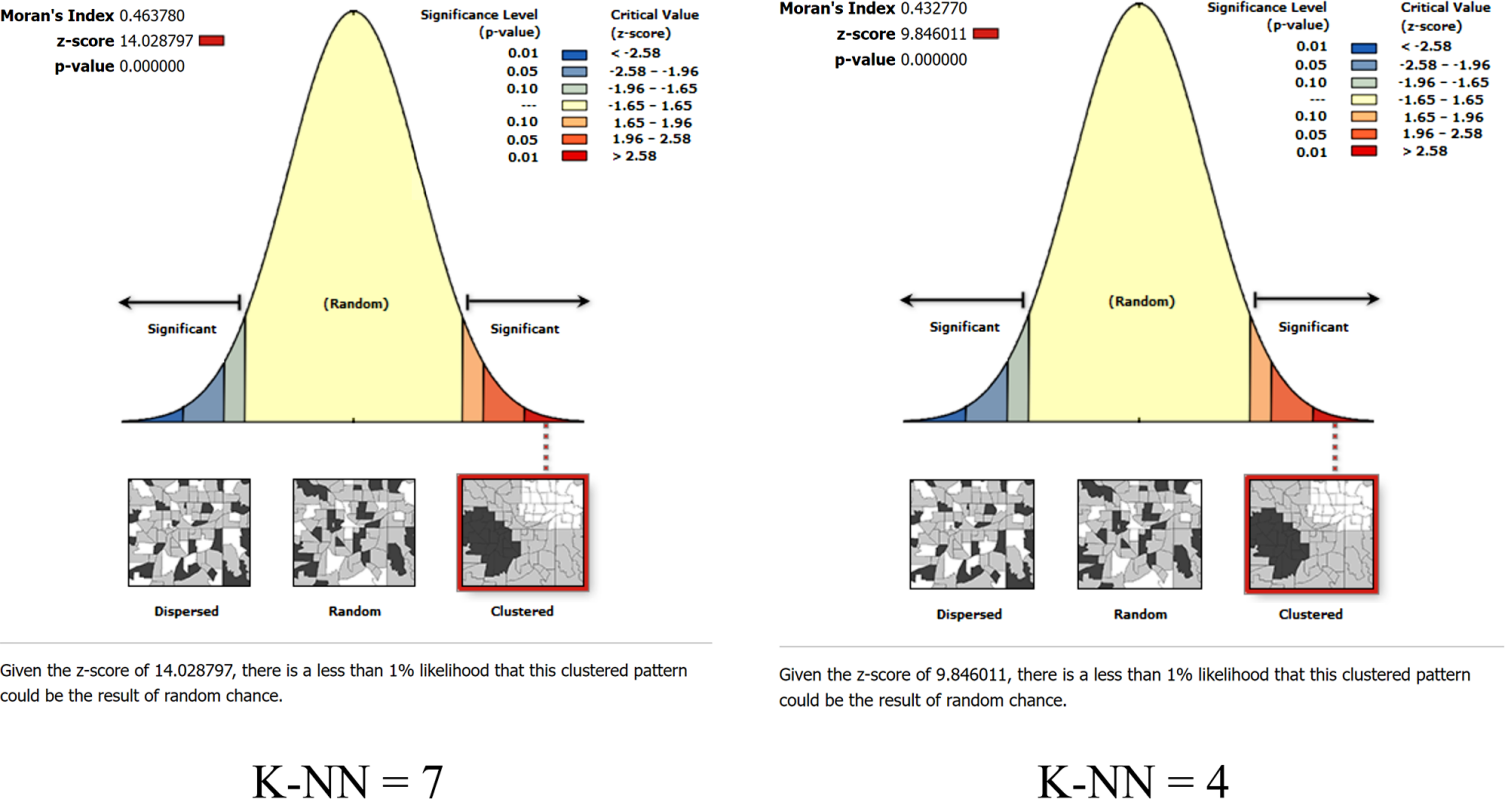
Global Moran’s I of TB incidence in 2021

Smaller values of K-NN (K-Nearest Neighbors) will make the model more sensitive and larger values of K-NN may lead to underfitting. The stability of the spatial autocorrelation results can be assessed by comparing Moran’s I at different K-NN values. If two different K-NN values give similar results, then the result can be considered stable. Therefore, we chose a smaller value (K-NN = 4) and a larger value (KNN = 7) as the spatial weights to compute Moran’s I separately to analyze the characteristics of the spatial distribution of TB incidence in 2021. When the spatial weight is set to the K-NN (K-Nearest Neighbors) method with 7 neighbors, the Global Moran’s I is 0.463, representing the existence of spatial autocorrelation. After 999 Monte Carlo simulations, the P-value is 0.000 and the Z-value is 14.028, indicating at least 99.9% confidence level. When the spatial weight is set to the K-NN method with 4 neighbors, the Global Moran’s I index is 0.432. After 999 Monte Carlo simulations, the P-value is 0.000 and the Z-value is 9.846, indicating a 99.9% confidence level, as shown in Fig. 4.

**Fig. 5:**
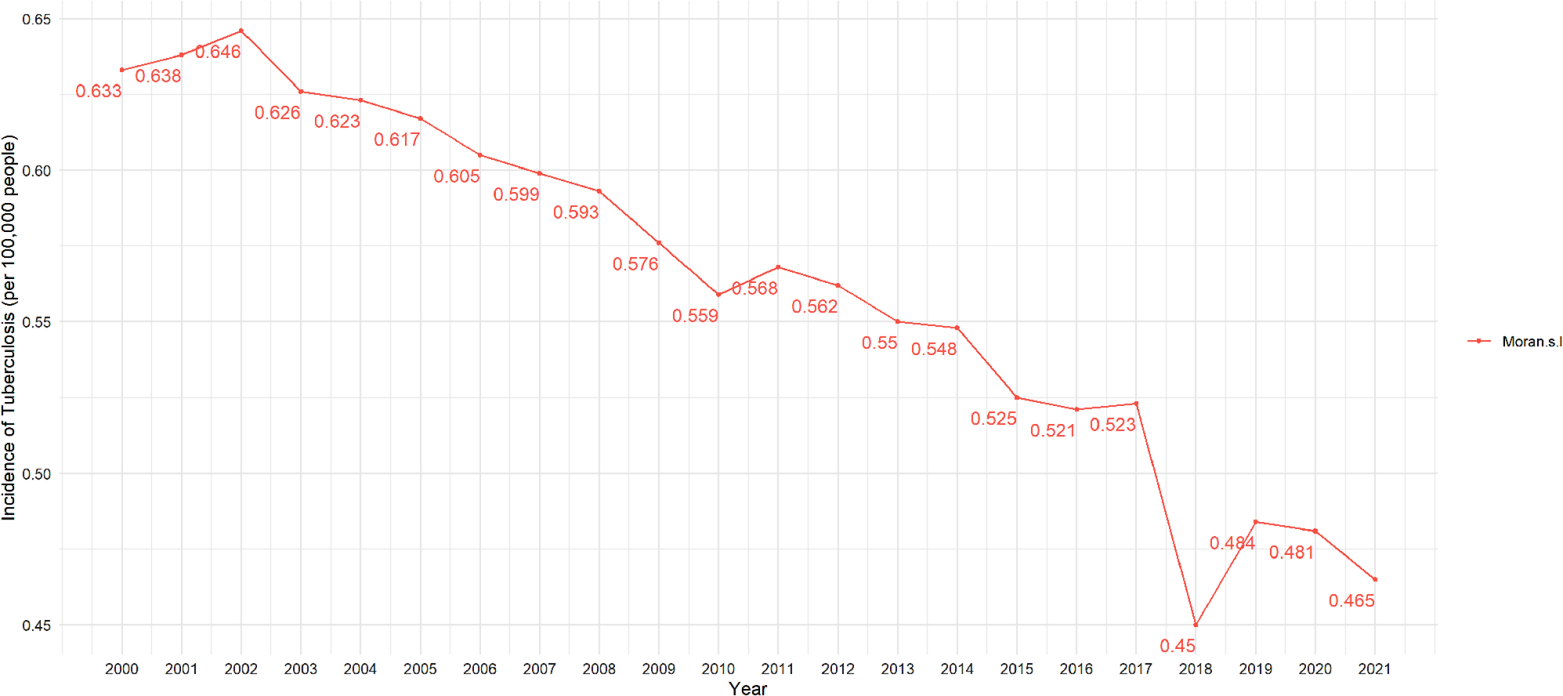
Global Moran’s I of TB incidence from 2000 to 2021

The above results further demonstrate the significant spatial autocorrelation of TB incidence in 2021. In addition, from 2000 to 2021, there is a general downward trend in the Moran’s I of global TB incidence, as shown in Fig. 5.

**Fig. 6:**
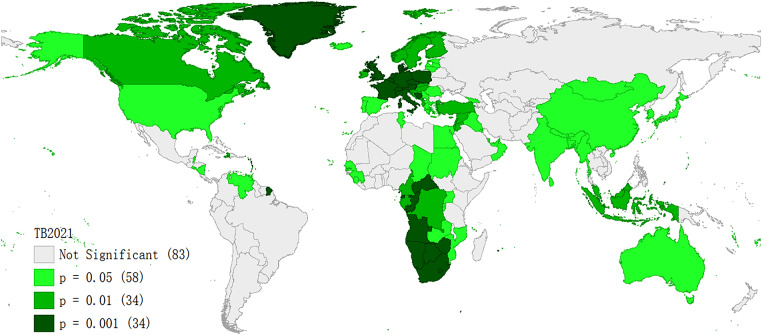
Significance map of local Moran’s I of TB incidence in 2021

Using 2021 TB incidence data to analyse the local Moran’s I, when the spatial weight is set to the K-NN method with 7 neighbors, local Moran’s I analysis revealed significant spatial clustering of TB incidence in most areas (P ≤ 0.05) as shown in Fig. 6.

**Fig. 7:**
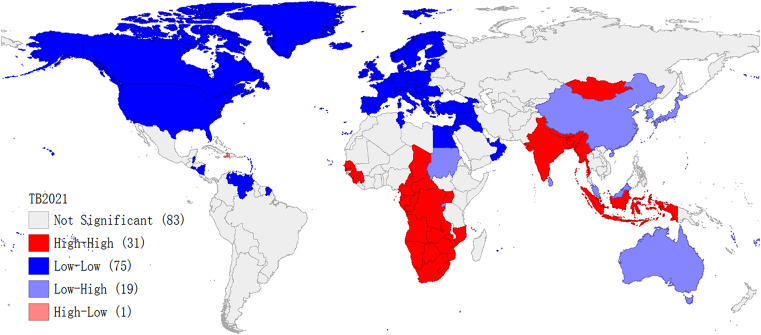
Cluster map of local Moran’s I of TB incidence in 2021

Most areas in North America and Europe were categorized as low-low clusters. Low-low clusters zone indicates that the incidence of TB is low both in the region and in the areas adjacent to it. Most areas in Central Africa, South Africa and East Africa, Southeast Asia, and South Asia were classified as high-high clusters, high-high clusters area indicates a high incidence of TB in both the area and neighbouring areas. Most areas in East Asia and Oceania were identified as low-high clusters, low-high clusters indicate a low incidence of TB in the area and a high incidence in its immediate area. Haiti is a high-low cluster area, with the high-low cluster indicating a high incidence of TB in the area and a low incidence in the area adjacent to it. It is also known that most countries and regions fall into the low-low cluster region, followed by those in high-high cluster region, with only one country falling into the high-low cluster region (Haiti), as shown in Fig. 7.

### Regression analysis results

**Fig. 8:**
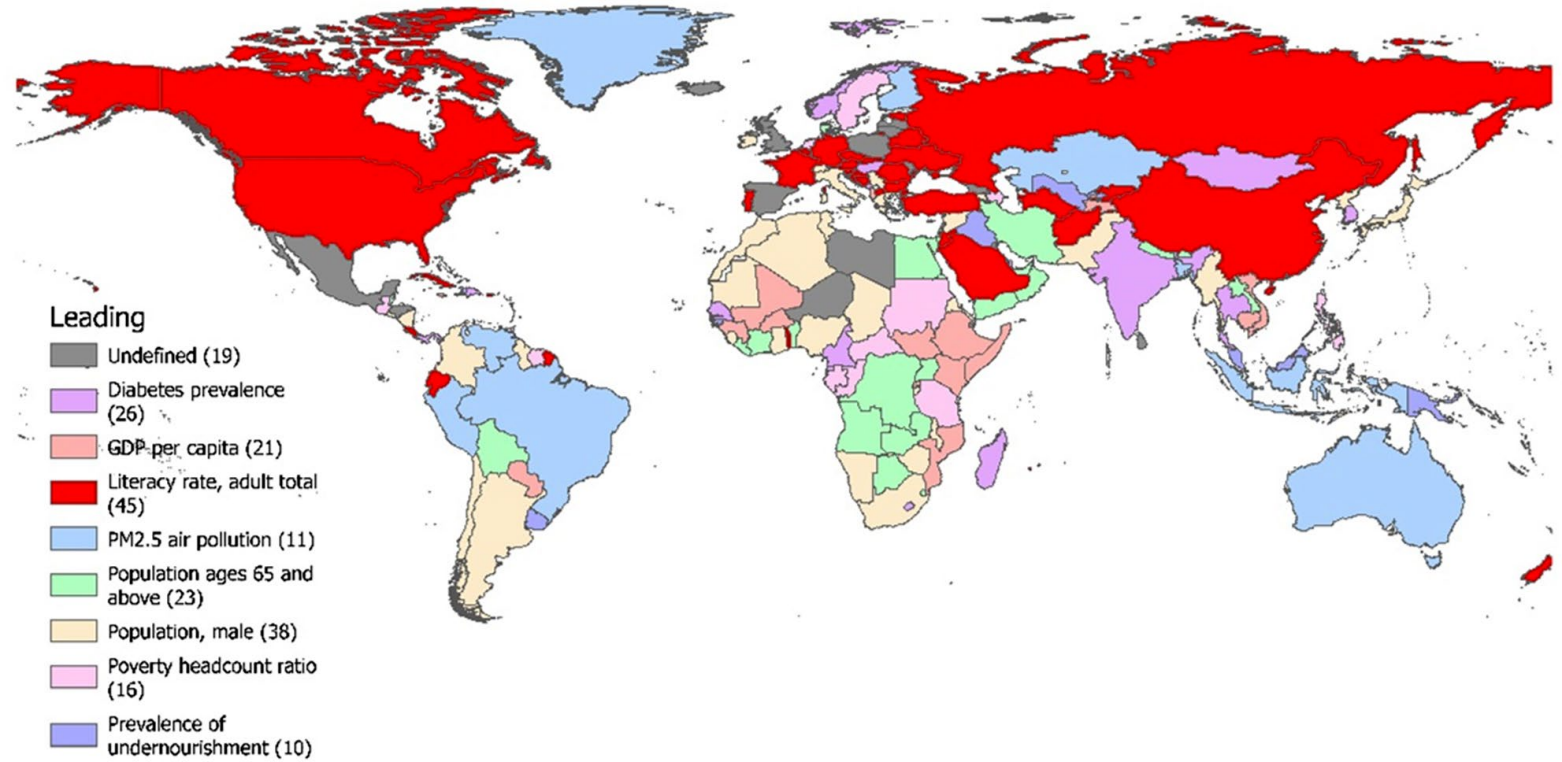
Analysis of the leading risk factors for TB

Figure 8 shows the leading risk factors for TB incidence in each country and region by comparing the regression coefficients from multiple linear regression analysis. The leading risk factor affecting the largest number of countries and regions is literacy rate, followed by the proportion of the male population, while PM_2.5_ air pollution and prevalence of undernourishment were the leading risk factors for TB affecting the least number of countries and regions. In South Africa and Central Africa, the leading risk factors for TB incidence were mainly the population ages 65 and above and the population of males. In Europe (including Russia) and North America, the leading risk factor for TB incidence was mainly the literacy rate. In other areas, the leading risk factors vary considerably among neighbouring countries and regions. An additional table shows regression coefficient in more detail [see Additional file 1].

**Fig. 9:**
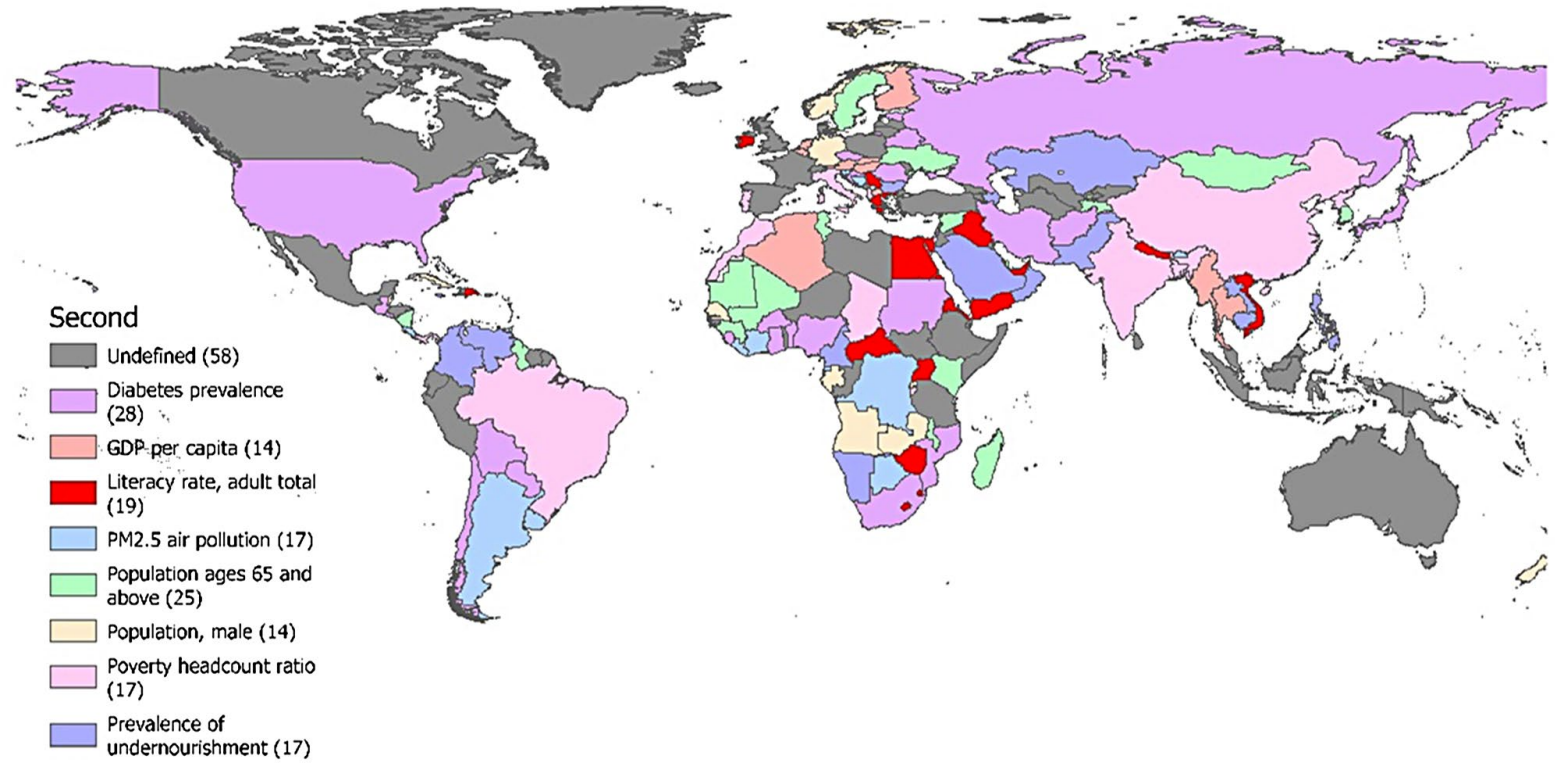
Analysis of the second leading risk factors for TB

Figure 9 shows the second leading risk factor for TB incidence in each country and region by comparing the regression coefficients from multiple linear regression analysis. The second leading risk factor for TB incidence affecting the largest number of countries and territories was the prevalence of diabetes, followed by the population ages over 65 and above. The population of males and GDP per capita were the second leading risk factors that affect the incidence of TB in the fewest countries and regions. Overall, even in neighbouring countries and regions, there are relatively large differences in the second leading risk factor for TB incidence.

**Fig. 10:**
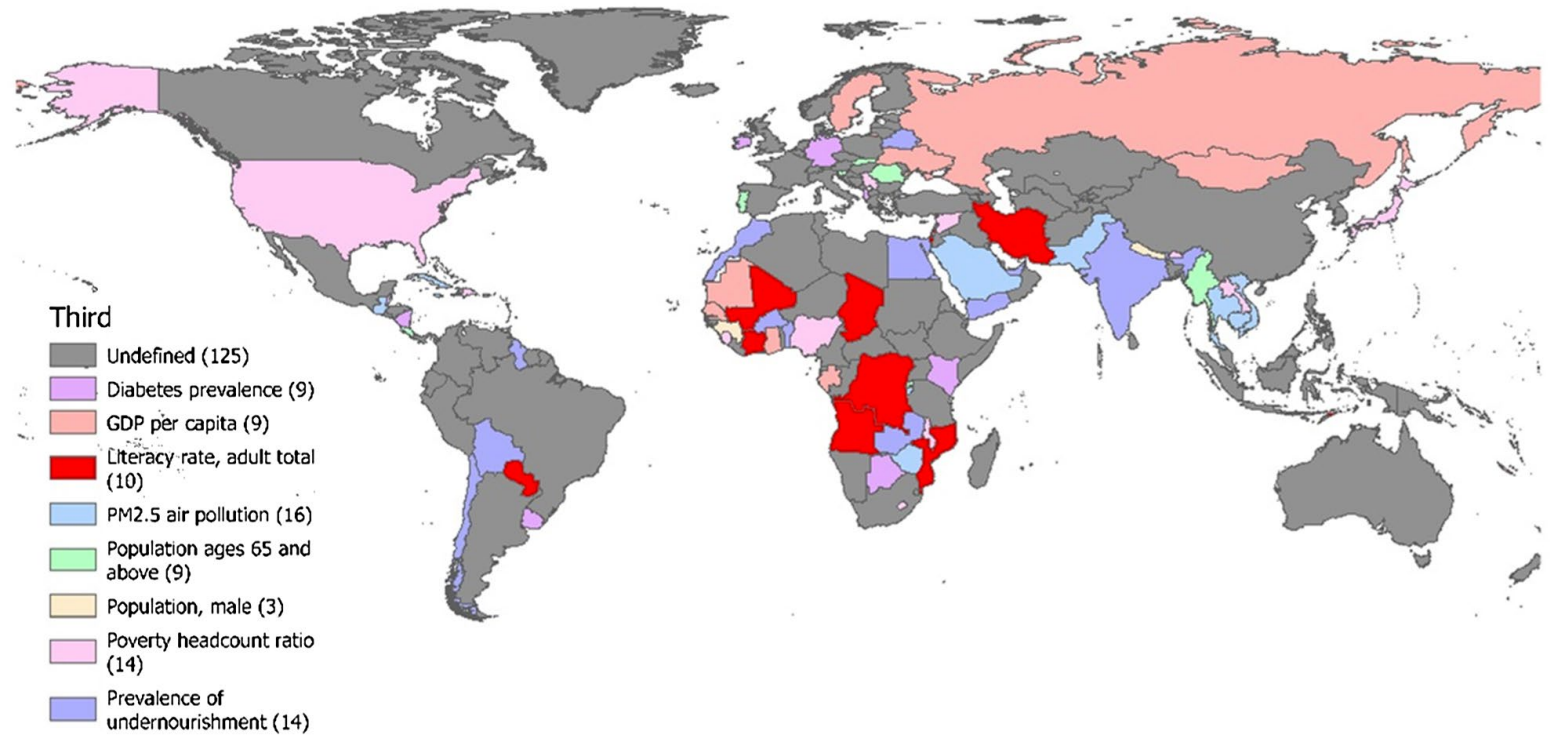
Analysis of the third leading risk factors for TB

Figure 10 shows the third leading risk factor for TB incidence in each country and region by comparing the regression coefficients from multiple linear regression analysis. The third leading risk factor for TB incidence affecting the largest number of countries and territories was PM2.5 air pollution, followed by the prevalence of undernourishment and poverty headcount ratio. The population of males was the third leading risk factor that affected the incidence of TB in the fewest countries and regions.

**Fig. 11:**
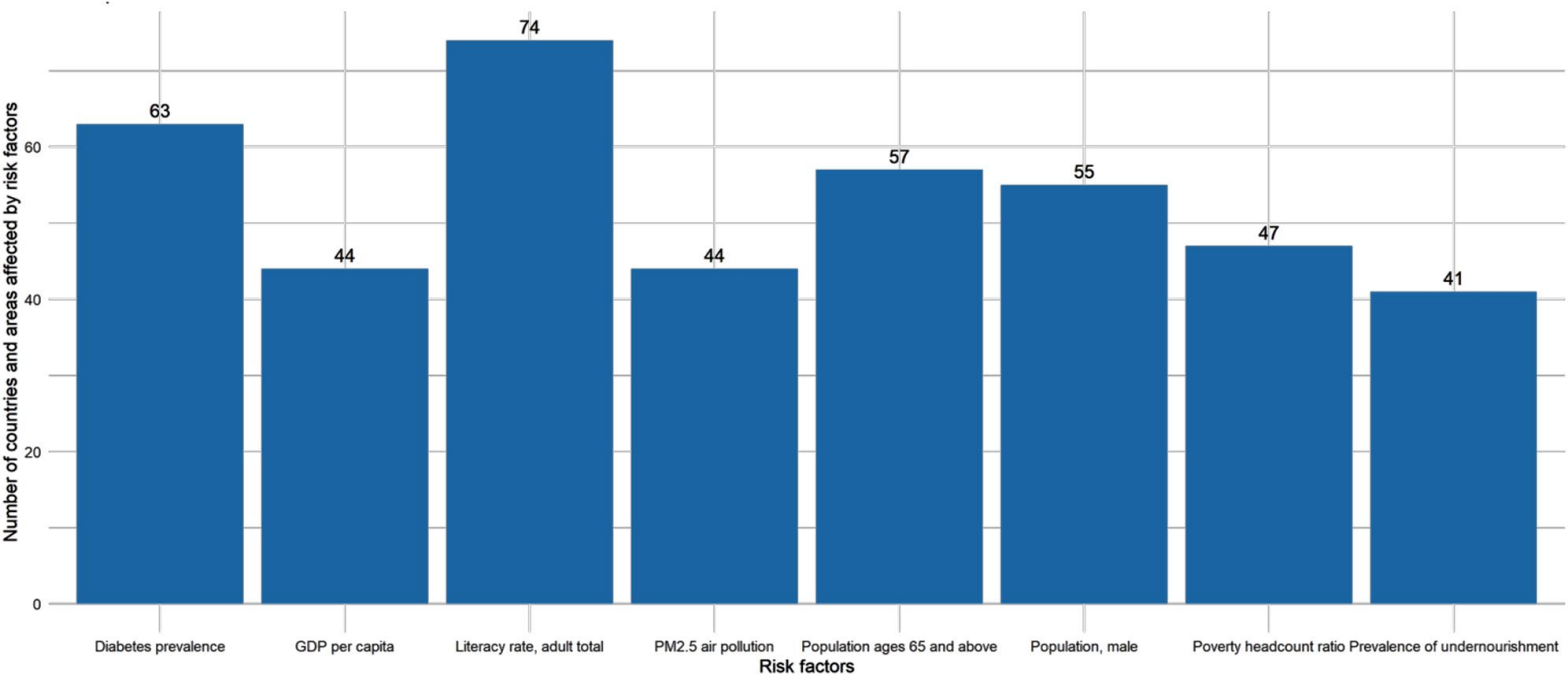
Analysis of the main risk factors for TB

Of the three leading risk factors for each country and region globally, the most frequent risk factor is literacy rate, followed by diabetes prevalence and population ages 65 and above, and the least frequent risk factor is the prevalence of undernourishment, as shown in Fig. 11.

### ARIMA model prediction results

**Fig. 12:**
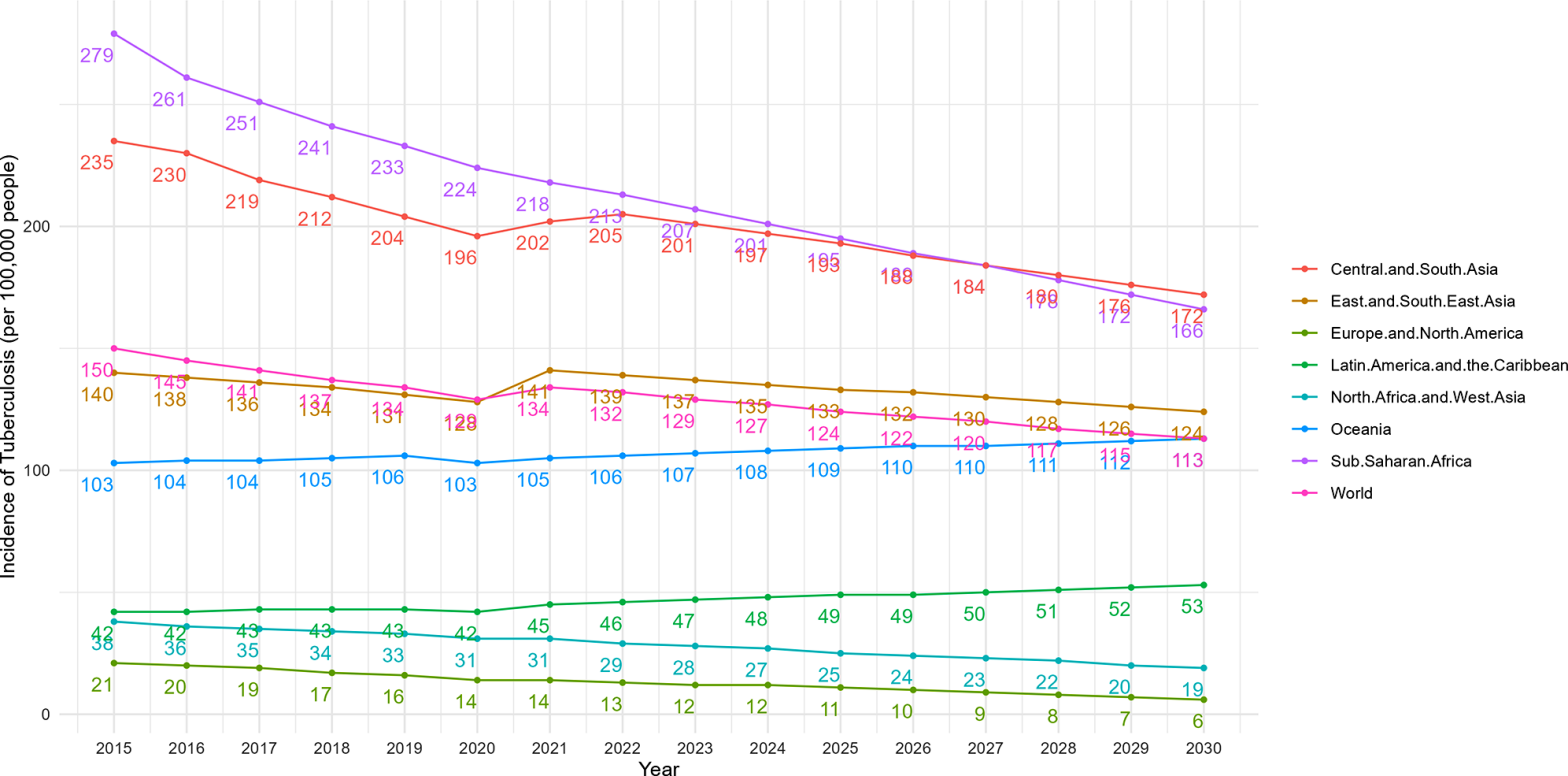
Projections of global and region category in TB incidence to 2030

According to the projections, the incidence of TB will decrease to varying degrees in 2030 compared to 2015 in all regions except Oceania, Latin America and the Caribbean, north Africa and west Asia showed the largest decline of 50%. However, the WHO Stop TB Strategy sets targets for 2030 of 80% decrease in TB incidence compared to 2015, but projections from the ARIMA model suggest that this target will be difficult to achieve in any region, as shown in Fig. 12.

**Fig. 13:**
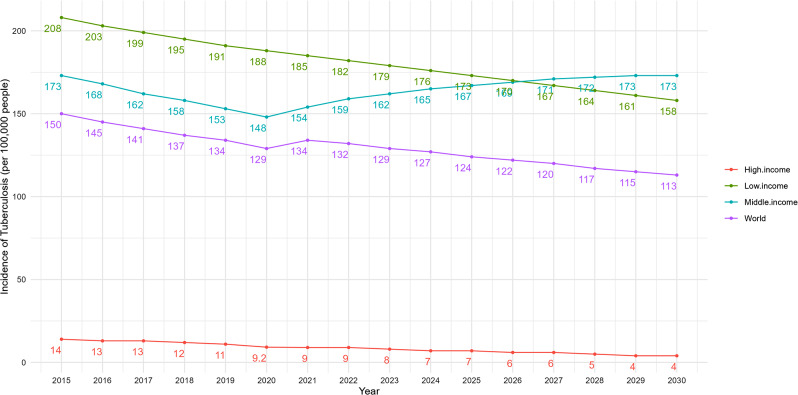
Projections of Gl obal and income category in TB incidence to 2030

According to the projections, the incidence of TB in 2030 will decrease to varying degrees compared to 2015 in all regions by income except middle-income countries and regions, high income countries and regions showed the largest decline of 71%. However, groups of countries and regions at any income level are still unlikely to meet the targets set by the WHO End TB Strategy of reducing TB incidence by 80% in 2030, as shown in Fig. 13.

## Discussion

The prime focus of this study was to investigate the global trend of TB incidence and underlying risk factors between 2000 and 2021. This study found that there was a trend of clustering in the spatial distribution of tuberculosis incidence, but that the trend of clustering was decreasing from year to year. There are differences in TB incidence across countries and regions with different income levels, this finding is in line with previous research, which has revealed that a vast majority of individuals with TB are concentrated in low- and middle-income countries (LMICs), underscoring the strong association between TB and poverty, as well as other socioeconomic risk factors [45]. This may also be linked to the truth that people in LMICs do not have enough money to choose better health care services. The most important finding of this study is that the risk factors for tuberculosis vary across countries and regions, with literacy rate being the risk factor with the relatively widest and deepest impact. Based on the results of the projections, with the present trends, the World Health Organization’s goal of ending the tuberculosis pandemic by 2030 is unlikely to be achieved, which is worthy of our attention.

Studies have shown that there is a higher incidence of TB not being diagnosed in a timely manner in LMICs, as a significant number of TB patients in LMICs seek primary treatment from private medical institutions, drug suppliers and lower-level public health institutions that do not have access to TB diagnostic services, which contributes to the this has led to the further spread of the epidemic [46, 47]. At the same time, health care professionals in LMICs are at an increased risk of TB infection, which can contribute to the epidemic’s spread [48, 49]. However, although the incidence of TB in LMICs is at a higher level compared to high-income countries, it is also generally declining, suggesting that previous TB control strategies in LMICs have been effective.

This study found a high geographic spatial autocorrelation in the spatial distribution of TB incidence even though the incidence was declining, suggesting that TB patients may be more clustered in specific areas. In previous studies, Africa (particularly sub-Saharan Africa) and Southeast Asia have generally been considered to be the regions with the most severe TB epidemics [13]. In this study, we can see that in addition to the high incidence areas in Africa and Southeast Asia, most of South Asia and Mongolia in East Asia are also areas of high TB incidence, which is a slight departure from the previous view. These countries and regions not only have a high TB burden of their own, but also tend to spread to surrounding countries or regions and should be given priority attention. On the other hand, significant geographical inequalities in TB incidence have been observed, with the majority of countries with high concentrations being low- and middle-income countries, and economic polarization contributing to clustering of TB incidence. Studies have shown that one-third of people diagnosed with TB in the Republic of South Africa do not start treatment or are not informed of their disease, while the rising proportion of extensively drug-resistant TB may also be contributing to the spread of the TB epidemic in South Africa [50, 51]. In addition, the number of cases of TB in South Asia is staggering yet has been under-appreciated. With a large, chaotic and unregulated private health sector, South Asia is also vulnerable to natural disasters and political disruption. When disaster inevitably strikes, emergency measures can greatly reduce the opportunistic spread of diseases such as TB [52].

In contrast, 19 countries in low-high cluster areas, which are surrounded by high prevalence areas but have managed to maintain low prevalence rates themselves, have TB control strategies that deserve further study and replication. In previous studies, Sudan has often been considered a country with a high TB burden [53]. However, this study shows that Sudan is in a low-high cluster area with a lower incidence of TB than the surrounding countries, suggesting that its TB control strategies are achieving some success. Of course, it is also argued that this is due to a lack of data management and higher levels of surveillance due to the conflict that has erupted in Sudan in recent years [54]. Rwanda, a low-high cluster country in Africa, on the other hand, has an effective TB surveillance system with precise, comprehensive, and both inside and outside consistent data that provides an excellent summary of the country, and TB control strategies developed through this system have been effective in decreasing the incidence of TB in Rwanda [55].

It is worth noting that although the incidence of TB in Oceania is still below the world average, and Fiji and Vanuatu are among the low-high cluster areas, the growing trend in the overall incidence of TB in Oceania is not negligible and requires attention [56]. The low-low cluster areas are mostly high-income countries and territories in Europe and the USA, which are most likely to be the first to achieve the 2030 complete elimination plan but should also be aware of the TB risks associated with migration from high-incidence countries [57].

Countries and regions with identical leading risk factors, comparable cultural affinities, and geographic proximity may opt for analogous TB control strategies. It is noteworthy that several neighboring countries in Central Africa have a population ages 65 and above as the leading risk factor for TB. This phenomenon can be attributed to the fact that although the degree of population aging in Africa is relatively low, this is due to a comparatively young age structure, high rates of fertility and death in the African population. However, elderly individuals in the African region generally experience a higher burden of chronic diseases and infectious diseases due to healthcare conditions, nutritional status, and other factors. This view is supported by relevant research, which indicates that immune function decreases with age, and the disease burden of elderly individuals in Africa is increasing [58].

In high-income and low TB incidence areas such as Europe and North America, low literacy rates have become a leading risk factor for TB incidence [59]. Low literacy rates may lead to low health literacy, which is detrimental to public access to health education. Europe and the United States have received a large influx of immigrants, whose health literacy is relatively poor, and who have also brought new burdens of TB [60, 61]. Therefore, it is necessary to implement TB interventions targeted at immigrants [62–66].

From the perspective of the second leading risk factor, there is low similarity among neighboring countries and regions. However, countries that share the same leading risk factor and second leading risk factors can be regarded as homogeneous countries and adopt similar TB prevention and control measures. Considering the third leading risk factor, our study found that many countries and regions have multiple risk factors, especially those with a high incidence of TB, such as Africa and Southeast Asia. This suggests that the incidence of TB in these areas is a complex issue, influenced not only by a single factor but by multiple factors.

In these areas with a high incidence of TB, comprehensive interventions need to be developed, including improving people’s health literacy, improving the living environment and strengthening health services. In particular, interventions targeting these risk factors are necessary [67]. For example, for areas with a high proportion of men, we need to strengthen health promotion and education for men and encourage them to undergo health screening and preventive measures [68]. There is the need for regular screening and treatment in the community and appropriate medical services and support. In addition, we need to further strengthen disease surveillance and data collection in order to better understand and control the spread of TB in different areas and groups.

This study demonstrates that low literacy rates are one of the most common risk factors for the occurrence of TB. Literacy rates are closely related to education level. There is a significant link between education and health, and low levels of education may exacerbate health problems [69, 70]. Therefore, enhancing education plays an important role in improving public health and preventing TB transmission. Governments should increase investment in education and health to raise the standard of public health and decrease the occurrence and spread of TB [71]. The study also found that diabetes prevalence is the second most common risk factor for the occurrence of TB. Previous research has shown that diabetes increases susceptibility to TB [72].

In addition, the study also points out that the 65 years and older age group is the third most common risk factor for the occurrence of TB. This is because the immune system of older people declines and their body’s resistance weakens, making them more susceptible to various diseases. Furthermore, aged persons are more likely to have other chronic conditions, which raises the chance of TB infection [73, 74]. Therefore, the elderly should also pay attention to TB prevention, exercise regularly, maintain a healthy diet, and improve their body’s immunity. These risk factors have a widespread impact and should receive more attention from relevant authorities.

### Limitations of the study

This study has some limitations that should be acknowledged. Firstly, the data used in the study are not exhaustive, and several risk factors were not included in the analysis. Moreover, some of the data used in this study have a high number of missing values, which could potentially bias the results. In addition, although the data used in this study are from official sources and we believe that they reflect the real situation, some countries may also have some errors in their official data due to their more backward level of development, which may have an impact on the results. Additionally, it is important to note that TB incidence rates often exhibit spatial variations within countries and regions, with notable differences between rural and urban areas. To obtain more comprehensive and accurate results, it is essential to incorporate more detailed data that capture these spatially aggregated trends.

Furthermore, it should be recognized that TB in many countries and regions is influenced by a multitude of risk factors, and these factors can interact with each other, potentially leading to complexities and inaccuracies in the results. Future studies should aim to address this issue by utilizing more comprehensive data sets and further reducing the effect of multicollinearity.

## Conclusion

This study indicates that the literacy rate exhibits the most substantial influence on TB compared to other risk factors, impacting a significant number of countries and regions. Additionally, the literacy rate consistently emerges as one of the leading risk factors across all countries and regions. Notably, there exists substantial heterogeneity in the leading risk factors among different countries and regions. The findings of this study significantly contribute to our understanding of the global burden of TB and the spatial distribution patterns, breaking them down at national and regional levels. The model developed in this study holds the potential to assist policymakers in devising tailored interventions that are locally appropriate, thereby facilitating a more effective reduction in TB incidence and associated risk factors. Ultimately, such interventions can contribute to achieving the 2030 goal of ending the TB epidemic.

### Electronic supplementary material

Below is the link to the electronic supplementary material.

Supplementary Material 1: Regression coefficient

## Data Availability

The datasets supporting the conclusions of this article are available in the World Bank Open Data, https://data.worldbank.org/indicator.

## Abbreviations

TB: Tuberculosis
GNI: Gross National Income
IDW: Inverse Distance Weighted
Moran’s I: Moran’s Index
K-NN: K-Nearest Neighbors
AIC: Akaike information criterion
ARIMA: Autoregressive Integrated Moving Average
MLE: Maximum Likelihood Estimation
LMIC: Low- and Middle-Income Country
